# Interferon-gamma inducible protein-10 as a potential biomarker in localized scleroderma

**DOI:** 10.1186/ar4378

**Published:** 2013-11-13

**Authors:** Kelsey E Magee, Christina E Kelsey, Katherine L Kurzinski, Jonhan Ho, Logan R Mlakar, Carol A Feghali-Bostwick, Kathryn S Torok

**Affiliations:** 1 Division of Rheumatology, Children’s Hospital of Pittsburgh of the University of Pittsburgh Medical Center, Pittsburgh, PA 15224, USA; 2 Department of Dermatology, University of Pittsburgh School of Medicine, Pittsburgh, PA 15213, USA; 3 Division of Pulmonary, Allergy and Critical Care Medicine, University of Pittsburgh School of Medicine, Pittsburgh, PA 15213, USA

## Abstract

**Introduction:**

The purpose of this study was to evaluate the presence and levels of interferon-gamma inducible protein-10 (IP-10) in the plasma and skin of pediatric localized scleroderma (LS) patients compared to those of healthy pediatric controls and to determine if IP-10 levels correlate to clinical disease activity measures.

**Methods:**

The presence of IP-10 in the plasma was analyzed using a Luminex panel in 69 pediatric patients with LS and compared to 71 healthy pediatric controls. Of these patients, five had available skin biopsy specimens with concurrent clinical and serological data during the active disease phase, which were used to analyze the presence and location of IP-10 in the skin by immunohistochemistry (IHC).

**Results:**

IP-10 levels were significantly elevated in the plasma of LS patients compared to that of healthy controls and correlated to clinical disease activity measures in LS. Immunohistochemistry staining of IP-10 was present in the dermal infiltrate of LS patients and was similar to that found in psoriasis skin specimens, the positive disease control.

**Conclusions:**

Elevation of IP-10 levels in the plasma compared to those of healthy controls and the presence of IP-10 staining in the affected skin of LS patients indicates that IP-10 is a potential biomarker in LS. Furthermore, significant elevation of IP-10 in LS patients with active versus inactive disease and correlations between IP-10 levels and standardized disease outcome measures of activity in LS strongly suggest that IP-10 may be a biomarker for disease activity in LS.

## Introduction

Scleroderma, a connective tissue disease characterized by cutaneous sclerosis, is a broad term that encompasses both forms of the disease: systemic sclerosis (SSc) and localized scleroderma (LS), also known as morphea. SSc is a systemic disorder characterized by skin, vascular and visceral organ sclerosis, which more commonly affects adults. LS, which is more prevalent in children, is characterized by sclerosis that is typically limited to the skin, subcutis, and underlying bone and tissue without vascular or internal organ involvement. Although both SSc and LS share a common underlying pathophysiology of excessive production and deposition of collagen and sclerosis in an autoimmune setting, they are clinically different with unique morbidities and prognoses. LS has a different pattern of morphology and skin lesion distribution than SSc; it encompasses several subtypes including plaque morphea (circumscribed superficial), generalized morphea, linear scleroderma of the trunk/extremities or head, deep morphea (circumscribed deep), pansclerotic morphea and mixed morphea [1].

Though the dermatopathology of these entities is similar and sometimes difficult to differentiate, there are a few characteristics which dermatopathologists document as occurring more frequently in LS compared to SSc, such as more overlying epidermal atrophy, more intense inflammation and more diffuse dermal sclerosis [2]. Both LS and SSc share findings of an earlier active disease phase with newer lesions demonstrating a lymphocytic infiltrate with a variable number of plasma cells and eosinophils [2]. As lesions evolve, inflammation density decreases as collagen bundles thicken and skin sclerosis increases in the later fibrotic phase of the disease [2].

The inflammatory pathway of scleroderma has been associated with several cytokines and chemokines. In addition to playing a role in the physiological process of immune cell maturation and trafficking, chemokines induce, maintain and amplify inflammatory and immune reactions [3]. Previous reports suggest that cytokines of various T-helper cell lineages contribute to both forms of scleroderma, LS and SSc [4,5]. An inflammatory chemokine of interest in this study, interferon-gamma inducible protein-10 (IP-10, CXCL10), belongs to the CXC chemokine subfamily and is known to play a role in inflammatory responses in several autoimmune diseases, including systemic lupus erythematosus [6], juvenile dermatomyositis [7] and SSc [5,8]. IP-10 acts through CXCR3 receptors that attract Th-1-type lymphocytes to inflammatory sites in the skin and contributes to several skin diseases, including psoriasis [9]. Interferon-gamma (IFN-γ) stimulates the secretion of IP-10 from keratinocytes and other immune cells, including leukocytes, neutrophils, eosinophils, macrophages and monocytes, which induces an inflammatory response [3]. Activated Th cells, B cells, macrophages and NK cells express CXCR3 and are then attracted to the inflamed tissue areas by IP-10 [3], which may account for the inflammatory infiltrate present in the skin of scleroderma patients.

In regards to scleroderma, most studies to date have focused on IP-10 expression in SSc. IP-10 expression is elevated in the serum [5], plasma [8] and skin [10] of SSc patients compared to that of healthy controls, and levels reflect active disease [5,8]. Furthermore, recent studies in SSc have shown that elevated plasma IP-10 levels correlate significantly with the Medsger Severity Index for muscle, skin and lung involvement and thus act as a serological marker of disease severity [8]. However, the expression of IP-10 in the peripheral circulation and skin of LS patients has not been studied. Thus, this study was designed to evaluate IP-10 in the circulation and local tissue in LS patients, with the additional goal of comparing IP-10 to standardized disease activity parameters.

## Methods

### Study participants

The University of Pittsburgh Institutional Review Board (IRB) approved four separate protocols for (1) blood sample and clinical data collection of LS patients, (2) blood sample collection from de-identified healthy controls, (3) skin sample analysis of LS patients and (4) skin sample analysis of de-identified controls. Blood sample and clinical data collection of LS patients was performed through the National Registry of Childhood Onset Scleroderma (NRCOS) which has been approved by the University of Pittsburgh IRB since 2003. Consent was obtained from parents for their children’s participation in the NRCOS study and assent was obtained when appropriate. Healthy plasma control samples were obtained through an IRB approved protocol to collect and store discarded ‘waste’ samples resulting from well child visits at the University of Pittsburgh. The study team was not involved in this sample collection. After making a request to this protocol, an honest broker provided the study team with de-identified samples. Only basic demographic information associated with the healthy plasma was given to the study team, including age and sex.

For skin sample analysis, waivers of informed consent were granted by the IRB for two separate protocols; one to access retrospective LS patient skin specimens and one to access healthy and disease control samples. The IRB approved the waivers of informed consent after determining the protocols were minimal risk and the waivers did not adversely affect the rights and welfare of the subjects. Samples from LS patients were collected as part of clinical care, stored in the dermatopathology laboratory and accessed retrospectively by the study team. An honest broker was used to procure the control skin samples from the dermatopathology laboratory, which were given to the study team in a de-identified manner.

The study of IP-10 in the plasma of LS subjects was performed first and included subjects with both active and inactive disease states. Subjects from this study who had available skin biopsy specimens with concurrent clinical and serological assessment during the active phase of disease were included in the immunohistochemisty IP-10 staining study.

### Blood specimen collection and clinical data

All LS patients had clinic visits from 2003 to 2012, during which standardized demographic, clinical and laboratory data were collected. Research blood samples were obtained from subjects by venipuncture at routine clinical visits as part of their participation in the pediatric scleroderma registry at the Children’s Hospital of Pittsburgh. Plasma was separated from whole blood via density-gradient centrifugation at 4°C within four hours of blood collection and promptly stored in 200 μL aliquots at -80°C until experimentation.

At the concurrent visit in which plasma was obtained, standardized clinical LS outcome measures were collected, including two disease activity measures: physician global assessment of activity (PGA-A) and the modified Localized Scleroderma Skin Score (mLoSSI) [11]. The PGA-A is usually completed in conjunction with the mLoSSI, and both outcome measures were used to determine if the patient’s disease status at the time of sample collection was ‘active’ or ‘inactive’. The PGA-A is graded on a 100-mm analog scale and includes the following cutaneous variables: new lesions within the previous month, enlargement of existing lesion within the previous month, erythema/violaceous color at the border of lesion and skin thickening/induration at the border of lesion [11]. The mLoSSI includes the sum of three separate scores from the following domains: erythema (none, mild, moderate, severe), skin thickness (none, mild, moderate, severe) and new lesion/lesion extension (present, not present) [11]. A patient was classified as having active disease if they had a PGA-A greater than zero and a mLoSSI greater than zero. The only available demographic information obtained for the de-identified healthy plasma controls was their age and gender. These samples were obtained at well child evaluations at the University of Pittsburgh, and, therefore, should represent a similar population.

### Cytokine measurement

Plasma IP-10 levels (detection limit: 7.5 pg/ml to 10,000 pg/ml) were measured using the Luminex bead immunoassay system (BioRad, Hercules, CA, USA), according to the manufacturer’s instructions at the University of Pittsburgh Cancer Institute (UPCI) Luminex Core Facility. The plasma samples were undiluted and compared to a High PMT-Standard Dilution Series (BioRad, Hercules, CA, USA). To investigate the reliability of the samples included in the Luminex analysis, levels of IP-10 in the plasma of subjects (LS and healthy) were measured in duplicate. Inter-panel and intra-assay control plasma samples were included to ensure consistency across panels.

### Tissue specimen collection

All skin specimens were accessed retrospectively by the study team per approved IRB protocols. The LS subjects’ skin specimens of interest included those with plasma IP-10 analysis during the active state of disease (typically their first clinical visit to the scleroderma center) with concurrent clinical assessment performed within a few months of the biopsy. Both the biopsy and plasma specimens of these subjects (n = 5) were obtained before any systemic therapy was initiated. At our center, typically the dermatologists will obtain a skin biopsy to confirm the diagnosis and then quickly refer to the pediatric rheumatology service in Pittsburgh for further management. The skin specimens from pediatric LS, psoriasis and healthy subjects consisted of residual tissues from 4 mm punch biopsies obtained for clinical purposes. All of the skin biopsies were performed by dermatologists at the University of Pittsburgh Medical Center, not by the patient’s treating rheumatologist or member of the study team. Samples were processed and stored according to a standard clinical protocol with immediate immersion in formalin for 24 to 48 hours, followed by fixation in paraffin for long term storage. Once requested by the study team, the paraffin blocks were made accessible for immunohistochemistry (IHC) analysis.

### Immunohistochemistry

Immediately prior to experimentation, the paraffinized block specimens were sectioned into 6 μm sections and fixed onto glass microscopy slides. IHC was performed on serial skin biopsy sections. Tissue was de-paraffinized in xylene and rehydrated sequentially in ethanol. Antigen retrieval was carried out using a steamer and endogenous peroxidases were quenched to reduce non-specific background staining. The tissue was blocked using 5% goat serum 4% BSA blocking buffer according to standard protocol. The tissue was then analyzed for the presence and location of IP-10 staining. To decrease variability, during the analysis of each individual LS patient, a psoriasis disease control and healthy control specimen were studied in tandem. Psoriasis was chosen as a positive disease control because IP-10 staining has been demonstrated in the dermis, as has been shown by Giustizieri *et al*. [9].

For each subject, a section of tissue was incubated overnight in the primary antibody, polyclonal IP-10 (1:250; Amgen Inc., Thousand Oaks, CA, USA) and the second section of the corresponding tissue was incubated overnight in the rabbit immunoglobulin G (IgG) isotype control (1:1000; Vector Laboratories, Inc., Burlingame, CA, USA) to serve as an antibody control. Following the primary antibody incubation, all tissue sections were washed in PBS and incubated with secondary antibody, biotinylated anti-rabbit IgG (1:200; Vector), for 30 minutes. The tissue was washed in PBS and incubated in Avidin Biotin Complex (ABC) (Vector) and aminoethylcarbazole solution (AEC) (Life Technologies Corporation, Carlsbad, CA, USA) for detection purposes. Images were acquired using an Olympus Provis microscope (Olympus America Inc., Center Valley, PA, USA).

### Hematoxylin and eosin staining

To assess the presence and degree of cellular infiltration in the skin sections, hematoxylin and eosin (H & E) staining was carried out according to standard protocol in skin sections adjacent to those used for IHC.

### Data analysis

#### ***Luminex assay***

All analyses were performed using SPSS v. 20 (SPSS, Chicago, IL. USA). Mean or median was used to describe data where appropriate. To determine if variability was present in the Luminex results between duplicate samples, intra-class correlation coefficients (ICC) were calculated. ICC values were interpreted according to the following ranges: excellent reproducibility: 0.6 to 1.0, moderate reproducibility: 0.4 to 0.59, and poor reproducibility: <0.4 [12].

Non parametric analyses were employed to evaluate differences in IP-10 plasma levels between subject groups using Wilcoxon rank sum analyses (α level of 0.05). IP-10 plasma levels were compared between LS and healthy controls, as well as within LS between active and inactive disease (activity classification based on clinical parameters). The relationship of cytokines to clinical measures of disease activity (PGA-A and mLoSSI) were also examined using Spearman’s rho (nonparametric correlation). Correlations of 0.16 to 0.29 were considered weak to low, 0.3 to 0.59 moderate to low, 0.5 to 0.69 moderate, 0.7 to 0.89 strong, and 0.9 to 1 very strong. Finally, IP-10 levels within classification subgroups of LS were compared to determine any differences. Subgroup classification was based on the Padua criteria [1].

#### ***Tissue analysis***

The tissue was analyzed for the presence and location of IP-10 staining. To evaluate the general degree of inflammatory infiltrate in the tissue sectioned, a dermatopathologist (JH) also evaluated the H & E slides which corresponded to the IP-10 stained tissue per subject. He described the cellular infiltrate present and scored the degree of cellular infiltrate in three random high power fields (HPF) per LS tissue section to determine the average amount of inflammation per specimen (mild, moderate, severe). The degree of infiltration in each HPF was quantified according to the following scale: mild: 1 to 50 cells, moderate: 51 to 150 cells, severe: >150 cells.

## Results

### Plasma analysis of IP-10

Plasma analysis included 69 patients with LS and 71 healthy children. Demographic and clinical data were obtained and are summarized in Table 1. This sample of LS subjects is representative of other large cohorts of pediatric LS patients [13]. The ICC between duplicate Luminex samples was very high (ICC = 0.996) indicating excellent reproducibility of IP-10 levels and, thus, the average level of the two samples was used in all further analyses.

**Table 1:** Demographic and baseline characteristics of 69 localized scleroderma (LS) subjects and 71 healthy control subjects who were included in the plasma analysis

**Characteristics**	**LS patients**	**Healthy controls**
	**(number = 69)**	**(number = 71)**
	number (%)	number (%)
Female	46 (66.7)	38 (54)
Caucasian	63 (91)	unknown
	Median (IQR)	Median (IQR)
Age at plasma sample in years	12.5 (10.0 to 16.0)	3 (2.0 to 9.0)
Age at disease onset in years	9 (7.0 to 11.0)	---
LS subtype		
Linear scleroderma trunk/extremity	26 (37.7)	---
Linear scleroderma head (En coup de sabre / Parry Romberg Syndrome)	14 (20.3)	---
Mixed morphea	9 (13.0)	---
Generalized morphea	8 (11.6)	---
Plaque (circumscribed superficial) morphea	5 (7.2)	---
Deep (circumscribed deep) morphea	4 (5.8)	---
Pansclerotic morphea	1 (1.4)	---
Clinical measures of activity:	median (IQR)	
PGA-A	41.0 (29.0 to 62.0)	---
mLoSSI	6.0 (3.0 to 13.0)	---

IQR, interquartile range; mLoSSI, modified Localized Scleroderma Skin Score; PGA-A, physician global assessment of activity.

**Figure 1:**
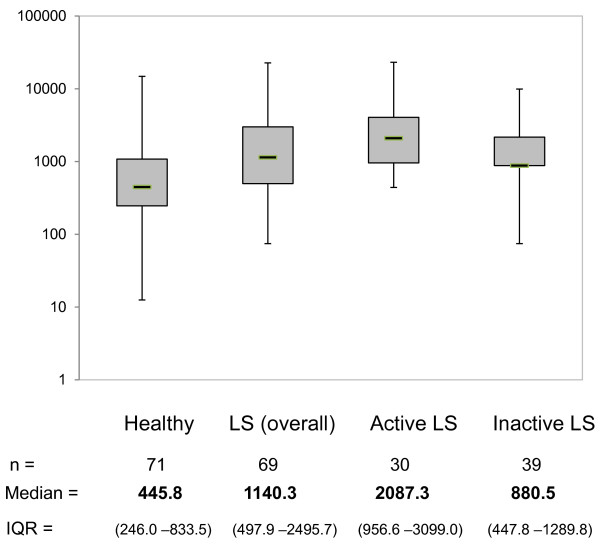
**IP-10 levels are increased in patients with localized scleroderma.** Plasma IP-10 levels in pediatric localized scleroderma (LS) patients, as a whole group, and then divided into active and inactive disease, compared to that of healthy controls. IP-10 levels in the plasma were significantly higher in pediatric LS patients in comparison to healthy controls, and those LS subjects with active disease had significantly higher levels than those with inactive localized scleroderma. IP-10, interferon-gamma inducible protein-10.

IP-10 levels in LS patients (median = 1,140.3 pg/ml, IQR = 497.9 to 2,495.7) were significantly elevated compared to those of healthy controls (median = 445.8 pg/ml, IQR = 246.0 to 833.5, *P* <0.001) (Figure 1). When disease activity status in LS patients was classified based on the above mentioned clinical parameters (mLoSSI and PGA-A greater than 0), 30 patients (43%) were considered to have active disease and 39 patients (57%) were considered to have inactive disease at the time of sample collection. Forty-two patients (61%) were on systemic therapy at the time of blood draw, with the majority being on methotrexate (36/42), either alone or in combination with prednisone. Three were on prednisone alone and there was one on each following: imuran, doxycycline and infliximab. The average duration of therapy for this group was 27.5 months. The remaining 39% (27/69) were not on systemic therapy at the time of blood draw; most of these patients (22/27) were in the active disease group (typically reflecting their first rheumatology visit).

IP-10 levels were significantly higher in active LS patients (median = 2,087.3 pg/ml, IQR = 956.6 to 3,099.0) compared to inactive LS patients (median = 880.5 pg/ml, IQR = 447.8 to 1,289.8, *P* = 0.001) (Figure 1). IP-10 levels in the plasma of LS patients had moderate to low, but statistically significant, correlations with scores of both clinical disease activity measures, the PGA-A (*r*_*s*_ = 0.45, *P* <0.001) and mLoSSI (*r*_*s*_ = 0.34, *P* = 0.004).

### Tissue analysis of IP-10

Of the 69 LS patients included in the luminex study, five had skin biopsies available which were taken within the 12 weeks (median 2 weeks, IQR 2 to 8 weeks) preceding their first rheumatology clinical visit, where the plasma and disease activity measures were obtained. These five subjects were determined as active based on their mLoSSI and PGA-A scores as well as being naive to systemic medication and were included in the IHC analysis of IP-10. The site of the biopsy for all patients was performed at the leading edge of a lesion (considered the most active site) and the lesion itself was considered active by both the dermatologist and rheumatologist. Clinical activity scores at the time of first rheumatology visit/blood draw were relatively high with a median PGA-A of 48 and a median mLoSSI of 7 (see Table 2 for clinical features). Plasma IP-10 levels of these five subjects reflected the active subgroup of LS subjects (described above) with a median and IQR of 2,814 (745 to 3,081) pg/ml (Table 2). Skin specimens were also obtained from two healthy controls, a Caucasian male, age 18, and an African American female, age 17 (Table 2). Two psoriasis disease control skin samples from a 47- and a 34-year-old woman were examined (Table 2).

**Table 2:** Demographics, clinical activity measures and IP-10 analysis of localized scleroderma (LS) patients and controls (psoriasis and healthy) participating in tissue analysis

**Clinical and laboratory features of tissue analyses subjects**	**LS subjects**	**Healthy controls**	**Psoriasis controls**
	**(number = 5)**	**(number = 2)**	**(number = 2)**
Demographic information	Number (%)	Number (%)	Number (%)
Female	4 (80)	1 (50)	2 (100)
Ethnicity:			
Caucasian	5 (100)	1 (50)	2 (100)
African American	---	1 (50)	---
Subtype:			
Linear (trunk/limb)	3 (60)	---	---
Plaque (circumscribed superficial)	2 (40)	---	---
Age	Median (IQR)	Ages in years	Ages in years
Age at onset, years	13 (6 to 16)	---	---
Age at biopsy, years	13 (10 to 16)	17, 18	34, 47
Tissue analysis	Number (%)	Number (%)	Number (%)
IP-10 staining present in cellular infiltrate	5 (100)	0 (0)	2 (100)
Degree of cellular infiltrate^a^			
Mild (1 to 50)	3 (60)	2 (100)	0 (0)
Moderate (51 to 150)	2 (40)	0 (0)	2 (100)
Clinical measures and plasma IP-10 of LS patients^b^	Median (IQR)		
PGA-A	48 (41 to 52)	---	---
mLoSSI	7 (7 to 8)	---	---
Plasma level of IP-10 (pg/ml)^c^	2,814 (745 to 3,081)	---	---

^a^Average cellular infiltrate in skin: out of three high power fields: 1 to 50 = mild; 51 to 150 = moderate; >150 severe; ^b^clinical and plasma measures obtained at first pediatric rheumatology visit, which was within 12 weeks of the biopsy date; ^c^determined by Luminex assay. IP-10, interferon-gamma inducible protein-10; IQR, interquartile range; mLoSSI, modified Localized Scleroderma Skin Score; PGA-A, physicians global assessment of activity.

**Figure 2:**
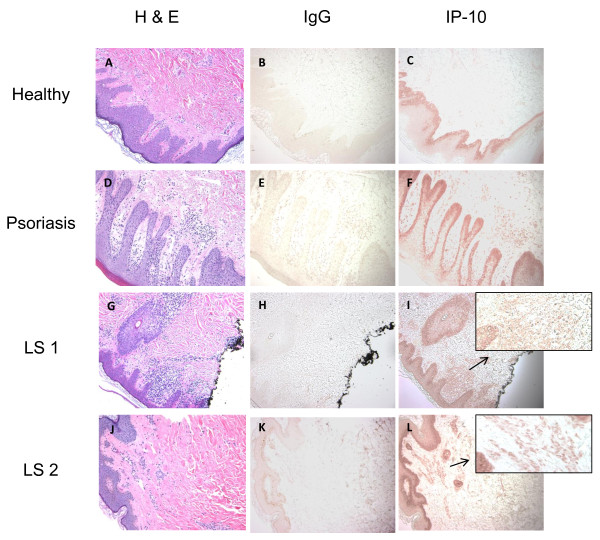
**IP-10 is identified in patients with localized scleroderma skin specimens, similar to psoriasis.** Immunohistochemical (IHC) analysis of IP-10 was performed on localized scleroderma, psoriasis (as positive control) and healthy patient skin specimens. All skin specimens were stained with hematoxylin and eosin (H & E) to visualize cellular infiltrate **(A, D, G, J)**. All specimens were also stained using an IgG control and demonstrated the absence of IP-10 staining for the control **(B, E, H, K)**. There was an absence of IP-10 staining in healthy controls represented by panel **C** (one illustrated out of two subjects). The psoriasis disease controls demonstrated moderate staining for IP-10, represented by panel **F** (one illustrated out of two subjects). Localized scleroderma (LS) patients demonstrated moderate IP-10 staining in the dermis, represented by panels **I** and **L** (two illustrated out of five subjects). IgG, immunoglobulin G; IP-10, interferon-gamma inducible protein-10.

When healthy human skin was stained for the expression of IP-10 no staining of the dermis was observed. In LS patients, IP-10 staining was observed in the superficial and deep dermal perivascular lymphoplasmacytic infiltrate (Figure 2). Examination of the two psoriasis skin biopsies demonstrated a moderate superficial perivascular and interstitial inflammatory infiltrate, consisting of lymphocytes and neutrophils, which stained positively for IP-10 (Table 2). The histological analysis of the H & E slides of the five LS patients by the dermatopathologist (JH) identified the presence of a mild-moderate degree of lymphoplasmacytic inflammation in all slides (Table 2).

## Discussion

This study demonstrated IP-10 to be significantly elevated in the peripheral circulation of pediatric patients with LS in comparison to healthy controls. This corresponds to findings in adult SSc studies in both Caucasian [10,14] and Japanese cohorts [5,15]. In addition, IP-10 was also present in the local tissue directly affected by LS. Finally, we found significant positive correlations between validated and prospectively collected disease activity parameters (mLoSSI and PGA-A) and plasma IP-10 levels, supporting a potential role of IP-10 in the active disease phase of LS. This is the first time IP-10 levels have been demonstrated to correspond to disease activity measures in LS. In adults, SSc studies have also demonstrated IP-10 in the plasma to correlate with disease activity and severity parameters, including skin thickness [8].

A greater understanding of the origin of IP-10 in skin sclerosis is required to develop effective therapies for LS. An increased expression of IFN-inducible chemokines (IP-10) is most likely a response to the high IFN-γ skin levels in early stages of disease [14]. IFN gene expression studies in SSc have shown that SSc patients have higher serum [14] and plasma IFN-inducible chemokine levels that correlate with microarray and qPCR IFN gene expression scores when compared with those of healthy controls [8].

In many Th-1-dominant autoimmune disorders, it has previously been determined that IP-10 and its receptor, CXCR3, play a role both in leukocyte recruitment to inflamed tissue and in the process of tissue damage [16]. Both LS and SSc are characterized by a predominant lymphocytic infiltrate during the active phase of the disease. The exact lymphocyte cell type has not yet been identified in LS, but studies in SSc demonstrate a mixture of CD8+ and CD4+ cells in affected skin [17]. It is possible that Th-1 cells, a subset of CD4+ T-helper cells, may be recruited locally to the site of the skin by IP-10. IP-10 belongs to the CXC chemokine family, known lymphocyte attractants, predominantly in areas of inflamed tissue [3]. In LS, the active phase, which has previously been shown to be induced by T cell-derived cytokines, is followed by a damage phase characterized by sclerosis [18]. The result of sclerosis and atrophy of the skin and underlying tissues may cause significant deformity and severe functional impairment [19]. Thus, it is essential to target potential pathogenic inflammatory proteins, such as IP-10, in the active inflammatory phase of LS to minimize cumulative, permanent disease damage.

To our knowledge, this is the first study which provides evidence of IP-10 as a possible biomarker for LS. However, this study is limited due to its cross sectional nature and small sample size, specifically for the results pertaining to skin samples. Also, the healthy control plasma samples were as a whole, younger than the LS patients. It would be interesting to evaluate if these results were replicable in a group of LS patients with age matched controls. A longitudinal approach of the peripheral blood IP-10 expression as disease status changes with systemic therapy is currently being undertaken by our study team and would assist in further defining IP-10’s role as a disease activity biomarker or predictor. Our ultimate goal in the investigation of the biologic contribution of IP-10 and the IFN pathway to disease activity will hopefully aid in the development of more directed and efficacious therapies.

## Conclusions

Our study indicates IP-10 as a potential clinically actionable biomarker in localized scleroderma by reflecting active disease, which may be useful for clinical management, such as medication response. Future directions include longitudinal analysis of peripheral IP-10 levels as the disease transitions from active to inactive states and later flares.

## Abbreviations

BSA: Bovine serum albumin; CHP: Children’s Hospital of Pittsburgh of the University of Pittsburgh Medical Center; H & E: Hematoxylin and eosin; HPF: High power fields; ICC: Intra-class correlation coefficient; IFN-γ: Interferon-gamma; IgG: Immunoglobulin G; IHC: Immunohistochemistry; IP-10: Interferon-gamma inducible protein-10; IRB: Institutional review board; LS: Localized scleroderma; mLoSSI: Modified localized scleroderma skin score; NRCOS: National registry of childhood onset scleroderma; PBS: Phosphate-buffered saline; PCR: Polymerase chain reaction; PGA-A: Physician global assessment of activity; SSc: Systemic sclerosis; UPCI: University of Pittsburgh Cancer Institute.

## Competing interests

The authors declare that they have no competing interests.

## Authors’ contributions

KM carried out the immunohistochemistry studies, analyzed and interpreted both experimental and clinical data and drafted and edited the manuscript. KK designed and carried out the immunohistochemistry and luminex studies and analyzed and interpreted both experimental and clinical data. JH assisted in securing skin specimens and analyzed the presence and degree of cellular infiltration in the dermis of skin specimens. CK performed the statistical analysis of clinical and laboratory data associated with the plasma samples and assisted in drafting and editing of the manuscript. LM assisted in the design and optimization of the immunohistochemistry assays, analyzed and interpreted experimental data and revised several sections of the manuscript. CFB participated in the design of the study, analysis of experimental data and coordination of the immunohistochemistry assays, and contributed to drafting and editing the manuscript. KT participated in the design and coordination of the studies, obtained and analyzed clinical data and drafted and edited the manuscript. All authors read and approved the final manuscript.
